# Endogenous Bioelectrical Modulation of Redox, Vascular Support, and Extracellular Matrix-Related Markers in Human Fibroblasts Following the REAC CO-IBZ Protocol

**DOI:** 10.3390/biomedicines14102164

**Published:** 2026-09-24

**Authors:** Sara Cruciani, Vania Fontani, Arianna Rinaldi, Giuseppe Garroni, Diletta Serra, Salvatore Rinaldi, Margherita Maioli

**Affiliations:** 1 Department of Biomedical Sciences, University of Sassari, 07100 Sassari, Italy; scruciani@uniss.it (S.C.); giugarroni21@gmail.com (G.G.); dilettaserra9@gmail.com (D.S.); mmaioli@uniss.it (M.M.); 2 Department of Reparative and Regenerative Medicine, Rinaldi Fontani Institute, 50144 Florence, Italy; vfontani@irf.it (V.F.); ari@irf.it (A.R.); 3 Research Department, Rinaldi Fontani Foundation, 50144 Florence, Italy

**Keywords:** fibroblasts, REAC CO-IBZ, redox regulation, vascular-support-related markers, gene expression, extracellular matrix, immunofluorescence, cell-cycle and stress response

## Abstract

**Background:** Fibroblasts are active regulators of tissue architecture, extracellular matrix organization, vascular support, oxidative balance, and stress-related cellular responses. The present study aimed to characterize the molecular response of human foreskin fibroblasts (HFF1) following exposure to the standardized Radio Electric Asymmetric Conveyer (REAC) Circulatory Optimization—Inside Blue Zone (CO-IBZ) protocol. **Methods:** Cells were exposed to nine standardized CO-IBZ sessions. RT-qPCR showed significant upregulation of *SIRT1*, *NOX4*, and *p16*, whereas *VEGF*, *p21*, and *HAS2* showed directional increases that did not reach statistical significance. Total antioxidant capacity, nitrite levels, and intracellular ROS showed small non-significant changes. Quantitative immunofluorescence analysis showed significant increases in *NOX4*, PARP1, and vWF fluorescence intensity, whereas *SIRT1* and collagen type III exhibited directional, non-significant increases. **Results:** Taken together, the transcriptional and biochemical findings, together with quantitative immunofluorescence measurements of protein markers, define a CO-IBZ-associated molecular profile involving redox-regulatory, vascular-support-related, cell-cycle- and stress-response-related, and extracellular matrix-related markers. **Conclusions:** These results complement previous in vitro characterizations of the REAC ACT-IBZ and MO-IBZ protocols and provide a basis for further investigation in primary cells and more complex biological models.

## 1. Introduction

Fibroblasts play a fundamental role in maintaining tissue structure, coordinating extracellular matrix remodeling, supporting vascular function, and integrating metabolic and stress-related signals within tissues. Far from being passive structural elements, fibroblasts are now widely considered dynamic regulatory cells capable of adapting their transcriptional state in response to environmental, mechanical, metabolic, oxidative, and inflammatory cues.

Because fibroblasts are widely distributed stromal cells that integrate redox regulation, vascular-support-related processes, and extracellular matrix organization, HFF1 cells were selected as a reproducible human model for the initial cellular characterization of the CO-IBZ protocol. This experimental model was used to define protocol-associated cellular responses, not to reproduce systemic physiology or predict clinical outcomes.

The integration of all these signals contributes to tissue resilience, reparative capability, and long-term cellular homeostasis [1,2].

Among the regulatory systems governing fibroblast adaptation [3], particular attention has been devoted to molecules that link oxidative balance [4], vascular regulation [5], cellular survival [6], and longevity-associated pathways [7,8]. Sirtuin 1 (*SIRT1*) is a NAD+-dependent deacetylase involved in multiple aspects of cellular maintenance, including DNA repair, oxidative stress control [9], metabolic regulation [10,11], and cellular lifespan extension [12]. Its expression is commonly increased in conditions of metabolic or oxidative distress, where it acts as part of a compensatory protective mechanism. At the same time, *SIRT1* participates in pathways related to mitochondrial function [13], redox adjustment, and cellular repair [14].

NADPH oxidase 4 (*NOX4*) plays an important role in controlled redox signaling [15]. Unlike other NOX isoforms predominantly associated with damaging oxidative bursts, *NOX4* has been increasingly recognized as contributing to regulated redox states, acting in several contexts as a modulator of endothelial function [16], cell migration, and differentiation dynamics [17]. Consistent with its role in regulated redox signaling, *NOX4*-mediated pathways may contribute to the induction of genes associated with cellular activation and vascular remodeling [18], including von Willebrand factor (vWF) [15]. Although vWF is classically considered an endothelial marker involved in angiogenesis and vascular homeostasis, it has also been implicated in wound healing and tissue remodeling contexts, including through its ability to bind growth factors such as *VEGF* [19]. In the present study, vWF was therefore considered as a vascular-related marker to be interpreted cautiously within the fibroblast model, without implying endothelial transdifferentiation [20]. In parallel, vascular endothelial growth factor (*VEGF*) is a critical regulator of vascular biology, contributing to endothelial support, tissue perfusion, angiogenic capacity, and oxygen supply, which are all key aspects of functional tissue maintenance [21].

Poly(ADP-ribose) polymerase 1 (PARP1) represents another important element in the cellular stress-response landscape [22]. Although it is essential for DNA repair, excessive activation of PARP1 consumes intracellular NAD+, potentially compromising mitochondrial function and cellular energy availability [23]. Thus, understanding how these molecular systems behave under different regulatory influences is crucial to gaining insight into fibroblast homeostasis and tissue adaptation.

Cell-cycle regulation and extracellular matrix homeostasis were further examined through *p16*, *p21*, and hyaluronan synthase 2 (*HAS2*). *p16* and *p21* were selected as context-dependent markers of cell-cycle and stress-response signaling. Although frequently investigated in relation to cellular senescence, neither marker alone is specific for a senescent phenotype, and their interpretation requires integration with additional molecular and functional evidence [24]. *HAS2* was included because of its role in hyaluronan synthesis, extracellular matrix organization, and fibroblast responses to environmental stress [25].

In parallel with biochemical and molecular regulatory systems, increasing attention has been directed toward the role of endogenous bioelectrical activity in cellular regulation [26,27]. Bioelectricity reflects the integrated contribution of ion channels, pumps, membrane potentials, and intercellular coupling, forming a complex regulatory code that contributes to cell behavior, proliferation, differentiation, and tissue patterning [27]. Consistently with this view, Radio Electric Asymmetric Conveyer (REAC) technology was developed with the specific rationale of interacting with altered endogenous bioelectrical states and contributing to their optimization. Previously published studies have documented REAC-induced modulation of gene expression profiles, cellular adaptive pathways, and structured reorganization phenomena in different biological systems, including direct cellular reprogramming models [28,29,30]. In the clinical domain, a recent prospective real-world observational study conducted within a Post-Market Clinical Follow-Up framework demonstrated that sequential application of REAC ACT, CO, and MO-IBZ protocols in patients with chronic low-grade inflammatory conditions was associated with clinically meaningful improvements in pain intensity, sleep disturbance, fatigue, and perceived well-being, with effects maintained at follow-up [31]. These observations provide a scientifically grounded precedent, indicating that controlled interaction with altered endogenous bioelectrical organization may be associated with structured cellular responses at the molecular and functional levels, thereby reinforcing the biological plausibility of the present investigation.

Previous in vitro investigations of REAC ACT-IBZ and REAC MO-IBZ in human fibroblasts have described protocol-specific molecular profiles related to inflammatory/longevity-associated and metabolic regulatory pathways, respectively [32,33]. Together with the prospective real-world PMCF study evaluating sequential ACT, CO, and MO-IBZ protocols in routine practice [31], these findings provide a translational framework for the present CO-IBZ investigation.

The aim of the present study was to characterize the molecular response of human foreskin fibroblasts (HFF1) following exposure to the standardized REAC CO-IBZ protocol. Specifically, we investigated transcriptional, biochemical, and protein-associated markers spanning redox-regulatory, vascular-support-related, cell-cycle- and stress-response-related, and extracellular matrix-related domains. This protocol-specific molecular characterization was designed to complement previous in vitro investigations of the REAC ACT-IBZ and MO-IBZ protocols [32,33] and to define the molecular profile associated with exposure of human fibroblasts to the standardized CO-IBZ protocol. The study was focused on molecular and cellular response patterns rather than on direct functional assessment of angiogenesis or on mechanistic bioelectrical measurements.

## 2. Materials and Methods

### 2.1. Cell Culture

Human foreskin fibroblasts (HFF1, ATCC^®^ SCRC-1041™) were obtained from the American Type Culture Collection (ATCC, Manassas, VA, USA) and cultured under standard conditions using Dulbecco’s Modified Eagle’s Medium (DMEM; Life Technologies, Carlsbad, CA, USA) supplemented with 10% fetal bovine serum (FBS; Life Technologies, Carlsbad, CA, USA), 2 mM L-glutamine (Euroclone, Milan, Italy), and 1% penicillin/streptomycin (Euroclone, Milan, Italy), maintaining controlled environmental conditions of temperature, humidity, and CO_2_ levels. Treated and control cells were seeded and maintained in parallel under equivalent conditions. Confluence at the beginning of treatment and passage number were kept comparable across experimental conditions to minimize variability related to growth phase or phenotypic drift. Cells were allowed to reach appropriate confluence prior to treatment to ensure stable proliferative conditions and to limit growth-dependent differences in gene expression.

### 2.2. REAC Technology and Protocol Architecture

REAC technology uses standardized, device-embedded protocols delivered through an Asymmetric Conveyor Probe (ACP). Within the Inside Blue Zone framework, ACT-IBZ (anti-inflammatory cellular treatment), CO-IBZ (circulatory optimization), and MO-IBZ (metabolic optimization) are distinct, operator-independent protocols. They share the BENE mod 110 technological platform and the standardized IBZ session architecture but retain separate device-embedded codifications. These configurations are automatically implemented by the device and cannot be modified by the operator. The present study evaluated the cellular response specifically associated with the CO-IBZ protocol.

### 2.3. REAC CO-IBZ Treatment Protocol

HFF1 cells were treated with the REAC Circulatory Optimization—Inside Blue Zone (CO-IBZ) protocol using a BENE 110 device (ASMED, Scandicci, Italy). CO-IBZ is a preset, device-embedded protocol whose delivery parameters cannot be independently selected or modified by the operator. The treatment cycle comprised nine 30-min sessions administered over three consecutive days, with three sessions performed each day. Each subsequent session began one hour after completion of the preceding session. This temporal organization reproduced the standard clinical administration schedule of the CO-IBZ protocol. During each session, the Asymmetric Conveyor Probe (ACP) was maintained in direct contact with the cell culture for 30 min. The ACP functions as the asymmetric conveyance interface of the REAC device and does not deliver an exogenous electric current to the culture. Untreated control cultures were maintained and handled in parallel under equivalent culture and environmental conditions.

### 2.4. Gene Expression Analysis

Following completion of the treatment cycle, total RNA was extracted from control and treated fibroblasts using the RNeasy Mini Kit (Qiagen, Hilden, Germany). RNA concentration and purity were assessed spectrophotometrically. Quantitative real-time PCR (RT-qPCR) was performed to assess changes in *SIRT1, NOX4, VEGF, p16, p21* and *HAS2* expression levels using the Luna^®^ Universal One-Step RT-qPCR Kit (New England Biolabs, Ipswich, MA, USA) on a CFX Thermal Cycler (Bio-Rad, Hercules, CA, USA). *GAPDH* was used as the reference gene. Relative expression was calculated using the 2^−ΔΔCt^ method, assuming comparable amplification efficiencies between target and reference assays. *GAPDH* was selected as the internal reference based on its established use in human fibroblasts under comparable experimental conditions. Its Cq values showed no statistically significant difference in *GAPDH* expression between control and CO-IBZ-treated samples, supporting its use for normalization in the present study.

Experiments were conducted using five biological replicates per condition, each analyzed in technical triplicate. Technical replicates were averaged within each biological replicate before statistical analysis, and statistical comparisons were performed using biological replicates as the experimental unit. This approach was adopted to avoid pseudoreplication and to ensure that statistical inference reflected biological rather than intra-assay technical variability.

### 2.5. Immunofluorescence Analysis

Immunofluorescence microscopy was used to evaluate signals associated with *SIRT1*, *NOX4*, PARP1, vWF, and collagen type III following REAC CO-IBZ treatment. Cells were fixed, permeabilized, and incubated overnight at 4 °C under gentle agitation with primary antibodies against *SIRT1* (Cell Signaling, Danvers, MA, USA), *NOX4* (Santa Cruz Biotechnology, Dallas, TX, USA), PARP1 (Cell Signaling, Danvers, MA, USA), vWF (Santa Cruz Biotechnology, Dallas, TX, USA), and collagen type III (Abcam, Cambridge, UK). After washing, fluorescence-conjugated secondary antibodies (Life Technologies, Grand Island, NY, USA) were incubated for 1 h at 37 °C in the dark. Nuclei were counterstained with DAPI (1 μg/mL) (Thermo Fisher Scientific, Grand Island, NY, USA). Fluorescence images were acquired using a THUNDER Imager DMi8 microscope (Leica, Nussloch, Germany). Standardized exposure parameters, illumination intensity, and acquisition settings were maintained across samples to ensure comparability. Images were selected based on technical quality and representativeness rather than signal intensity. A standardized ImageJ/Fiji analysis procedure was applied consistently across control and treated samples. Quantitative immunofluorescence was expressed as mean fluorescence intensity, and measurements associated with each biological replicate were summarized as a single replicate-level value before statistical comparison. Data are presented as mean ± SD (*n* = 5 biological replicates). The primary antibodies were used at the following working dilutions: *SIRT1*, 1:100; *NOX4*, 1:500 from a 1:50 stock solution; PARP1, 1:800; vWF, 1:500 from a 1:50 stock solution; and collagen type III, 1:500. The corresponding fluorescence-conjugated secondary antibodies were used at 1:500.

The RT-qPCR and quantitative immunofluorescence panels provided complementary marker-level readouts rather than a complete set of matched transcript–protein measurements. *SIRT1* and *NOX4* were assessed using both methods, whereas the remaining markers provided distinct readouts within the vascular-support, cell-cycle and stress-response, DNA-damage-response, and extracellular matrix-related domains.

### 2.6. Total Antioxidant Capacity

Total antioxidant capacity (TAC) in the culture medium after REAC CO-IBZ treatment was measured using the Total Antioxidant Capacity Colorimetric Assay Kit (Abcam, Cambridge, UK). Briefly, 100 μL of Cu^2+^ working solution was added to each standard and sample well. The plate was incubated at room temperature for 90 min on an orbital shaker and protected from light, according to the manufacturer’s instructions. Absorbance was measured at 570 nm using a microplate reader (Akribis Scientific, Common Farm, UK). TAC for each sample was calculated as (Ts/Sv) × D, where Ts is the TAC amount in the sample well derived from the standard curve (nmol), Sv is the sample volume added to the well (μL), and D is the sample dilution factor. Assays were conducted using five independent biological replicates per condition, with technical replicates used to assess analytical consistency.

### 2.7. Nitrite Measurement

The Griess Reagent Kit for Nitrite Determination (Thermo Fisher Scientific, USA) was used to assess changes in nitric oxide (NO)-related nitrite levels after REAC CO-IBZ treatment. According to the assay protocol, 20 μL of Griess Reagent were added to 150 μL of nitrite-containing samples and incubated for 30 min at room temperature. A microplate reader (Akribis Scientific, Common Farm, Barrow, UK) was used to measure absorbance at 548 nm, and values were analyzed relative to the photometric reference sample. Assays were conducted using five independent biological replicates per condition, with technical replicates used to assess analytical consistency.

### 2.8. Intracellular ROS Measurements

Intracellular ROS levels were assessed using a CM-H_2_DCFDA fluorescence probe (Thermo Fisher Scientific, USA). The dye was reconstituted in DMSO according to the manufacturer’s instructions. Following treatment, cells were incubated with CM-H2DCFDA at a final concentration of 10 µM for 30 min at 37 °C in the dark. After incubation, the dye was removed, the cells were washed with phosphate-buffered saline (PBS), and fluorescence was measured in fresh PBS using a microplate reader (Akribis Scientific, Common Farm, Barrow, UK; excitation: 485 nm; emission: 530 nm). Assays were conducted using five independent biological replicates per condition, with technical replicates used to assess analytical consistency.

### 2.9. Statistical Analysis

Statistical analyses were performed using GraphPad Prism 9.0 software (GraphPad, San Diego, CA, USA). Data normality was assessed using the Shapiro–Wilk test. Comparisons between untreated control and REAC CO-IBZ-treated cultures were performed using an unpaired two-tailed Welch’s *t*-test. Biological replicates were considered as the experimental unit for all quantitative analyses. Technical replicates were averaged within each biological replicate before statistical analysis and were used exclusively to assess analytical consistency. Relative gene expression was calculated using the 2^−ΔΔCt^ method and is presented as fold change relative to untreated controls. Statistical comparisons were performed at the biological-replicate level using the replicate values generated by the RT-qPCR analysis after averaging the corresponding technical triplicates. Quantitative statistical analysis was also applied to immunofluorescence mean fluorescence intensity, total antioxidant capacity, NO-related nitrite levels, and intracellular ROS datasets. Representative immunofluorescence images are shown together with the corresponding quantitative analysis. Data are reported as mean ± SD. A *p*-value < 0.05 was considered statistically significant.

## 3. Results

### 3.1. REAC CO-IBZ Treatment Is Associated with Changes in Redox-, Cell-Cycle-, Vascular-Support-, and Extracellular Matrix-Related Gene Expression

Quantitative RT-qPCR analysis revealed significant upregulation of *SIRT1* (Figure 1A; *p* ≤ 0.05), *NOX4* (Figure 1B; *p* ≤ 0.001), and *p16* (Figure 1D; *p* ≤ 0.01) in REAC CO-IBZ-treated fibroblasts compared with untreated controls. *VEGF* (Figure 1C), *p21* (Figure 1E), and *HAS2* (Figure 1F) showed directional increases that did not reach statistical significance. These findings describe changes across redox-regulatory, vascular-support-related, cell-cycle- and stress-response-related, and extracellular matrix-related transcripts.

### 3.2. Oxidative-Response Readouts Following REAC CO-IBZ Treatment

Total antioxidant capacity (Figure 2A), NO-related nitrite levels (Figure 2B), and intracellular ROS fluorescence (Figure 2C) were evaluated in HFF1 following REAC CO-IBZ treatment. REAC CO-IBZ treatment was associated with small, non-significant increases in total antioxidant capacity and NO-related nitrite levels compared with untreated control cells. Intracellular ROS levels, assessed using CM-H2DCFDA, showed no substantial differences between treated and control cells, with only a slight non-significant increase observed in REAC CO-IBZ-treated samples. These measurements provide no evidence of a treatment-associated increase in the assessed oxidative-stress-related readouts.

### 3.3. REAC CO-IBZ Treatment Is Associated with Changes in Redox-, Vascular-Support-, and Extracellular Matrix-Related Protein Markers

Quantitative immunofluorescence analysis showed a directional, non-significant increase in *SIRT1* fluorescence intensity and a significant increase in *NOX4* fluorescence intensity in REAC CO-IBZ-treated HFF1 compared with untreated controls (Figure 3). *SIRT1* is a key regulator of redox homeostasis, whereas *NOX4* generates hydrogen peroxide (H_2_O_2_) as its primary product and can act as a regulated redox mediator in vascular adaptation and remodeling. The transcriptional increases in *SIRT1* and *NOX4*, together with the corresponding quantitative immunofluorescence measurements, are reported as concurrent changes in redox-related markers; they do not establish a coordinated mechanism or causal interaction [34].

Quantitative immunofluorescence analysis showed significant increases in PARP1 and vWF mean fluorescence intensity in treated cells compared with untreated controls (Figure 4; PARP1: *p* ≤ 0.01; vWF: *p* ≤ 0.0001). The PARP1 finding is reported as a change in the measured fluorescence signal; enzymatic activity and PARylation were not assessed. Similarly, the vWF finding represents a change in fluorescence intensity within the HFF1 fibroblast model and is not interpreted as evidence of endothelial differentiation or vascular function.

Quantitative immunofluorescence showed a directional, non-significant increase in collagen type III mean fluorescence intensity in REAC CO-IBZ-treated HFF1 cells compared with untreated controls (Figure 5). *HAS2* expression likewise showed a directional, non-significant increase. Given the established involvement of *HAS2* and collagen type III in extracellular matrix organization [35], these findings are reported as directional changes in extracellular matrix-related markers, and their functional significance remains to be determined.

## 4. Discussion

The present study characterized the molecular response of the HFF1 human fibroblast model to the standardized REAC CO-IBZ protocol. The main finding was a multidomain, protocol-associated molecular profile involving redox-regulatory, vascular-support-related, cell-cycle- and stress-response-related, and extracellular matrix-related markers. Significant transcriptional increases in *SIRT1*, *NOX4*, and *p16* were accompanied by directional, non-significant changes in *VEGF*, *p21*, and *HAS2* and by quantitative immunofluorescence changes in *SIRT1*, *NOX4*, PARP1, vWF, and collagen type III.

The concurrent changes in *SIRT1*, *NOX4*, and *p16* are considered together as components of a multidomain, CO-IBZ-associated molecular profile. Because these markers were assessed at a single post-treatment time point and no temporal, pathway-intervention, or activity analyses were performed, the data do not establish a causal sequence, direct pathway interaction, or synergistic network among them.

Within the redox-related component of this profile, *SIRT1* and *NOX4* showed significant transcriptional upregulation. *NOX4* mean fluorescence intensity also increased significantly, whereas *SIRT1* mean fluorescence intensity showed a directional, non-significant increase. The absence of significant changes in intracellular ROS, total antioxidant capacity, or nitrite levels provides no evidence of overt oxidative stress under the experimental conditions. *NOX4*-derived H_2_O_2_ can function as a regulated signaling mediator [15,36], whereas *SIRT1* participates in oxidative-stress and mitochondrial regulation [37], protective cellular responses [38], and DNA-damage signaling [39]. These established biological roles provide context for the concurrent marker changes but do not demonstrate that *SIRT1* counterbalanced *NOX4* or prevented ROS accumulation. Nitrite was assessed as an indirect readout of nitric oxide-related signaling; its non-significant change does not provide evidence of a functional vascular effect [40].

PARP1 has established roles in DNA repair [22] and cellular stress responses [23]. In the present study, however, the increase in PARP1 mean fluorescence intensity is reported strictly as a marker-level change; PARP1 enzymatic activity and PARylation were not assessed.

Within the vascular-support-related marker panel, a significant increase in vWF mean fluorescence intensity was accompanied by a directional, non-significant increase in *VEGF* expression [21,41]. vWF was included because of its established relevance to vascular and tissue contexts [19,20]. Within the HFF1 fibroblast model, these observations do not establish endothelial differentiation or a functional vascular effect. The vWF finding is therefore reported strictly as a CO-IBZ-associated change in mean fluorescence intensity within the studied fibroblast model.

The significant increase in *p16* expression, together with the directional, non-significant increase in *p21* expression, represents a change in cell-cycle- and stress-response-related markers. These markers are context-dependent and do not by themselves establish altered proliferation, cell-cycle arrest, or cellular senescence [24]. Because SA-β-galactosidase activity, proliferation kinetics, and cell-cycle distribution were not assessed, the present design cannot distinguish a stress-associated regulatory response from entry into senescence or another cell-state change.

Similarly, the directional, non-significant increases in *HAS2* expression and collagen type III mean fluorescence intensity are compatible with an extracellular matrix-related response, although their functional significance remains to be defined [25].

Viewed together with previous studies of the ACT-IBZ and MO-IBZ protocols [32,33], the present findings extend a series of separate protocol-specific molecular characterizations performed within the same REAC technological platform. ACT-IBZ has been characterized primarily in relation to longevity-associated and inflammatory markers, MO-IBZ in relation to metabolic regulatory markers, and the present study defines a CO-IBZ-associated profile involving redox-regulatory, vascular-support-related, cell-cycle- and stress-response-related, and extracellular matrix-related markers. These separate studies were not designed as a direct head-to-head comparison among the three protocols.

This protocol-specific framework positions the CO-IBZ-associated molecular profile within the broader characterization of standardized REAC protocols.

The findings should be interpreted within the targeted in vitro design. The study was designed as a protocol-specific molecular characterization, and the corresponding functional consequences were not assessed. Functional angiogenesis assays and additional mechanistic analyses were therefore outside the present experimental design. At the technical level, neither local field distribution nor endogenous bioelectrical activity was directly quantified.

Experiments were performed using a standardized human fibroblast cell line and five independent biological replicates per condition. Studies in primary fibroblasts from multiple donors could extend the assessment to inter-donor variability and broader generalizability, which were not endpoints of the present experimental design.

The dispersion observed in some endpoints may reflect variability among independently processed cultures and analytical measurements; the present design does not permit attribution to a specific source. Although the immunofluorescence signals were quantitatively evaluated by mean fluorescence intensity analysis, orthogonal protein-level validation was not performed.

## 5. Conclusions

The standardized REAC CO-IBZ protocol was associated with a molecular response in HFF1 human fibroblasts, characterized by changes in markers related to redox regulation, vascular support, cell-cycle and stress responses, and extracellular matrix organization. The combined transcriptional and biochemical findings, together with quantitative immunofluorescence measurements of protein markers, define a CO-IBZ-associated molecular profile and complement previous in vitro characterizations of the REAC ACT-IBZ and MO-IBZ protocols. Studies in primary cells and more complex biological models may extend the assessment of the functional relevance and generalizability of this response.

## Figures and Tables

**Figure 1:**
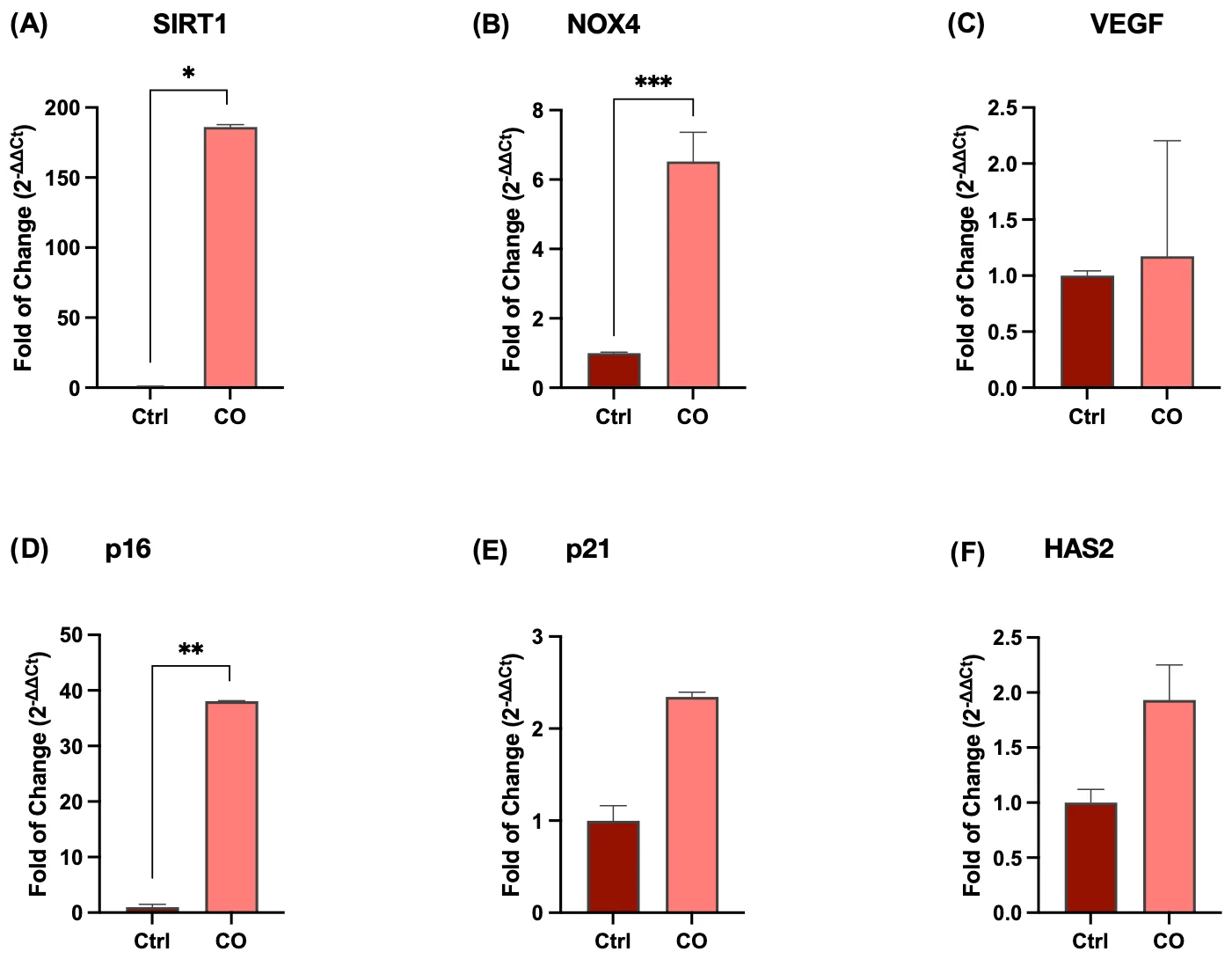
Gene expression of *SIRT1* (**A**), *NOX4* (**B**), *VEGF* (**C**), *p16* (**D**), *p21* (**E**), and *HAS2* (**F**) in control vs. REAC CO-IBZ-treated HFF1. The mRNA levels for each gene were normalized to glyceraldehyde-3-phosphate dehydrogenase (*GAPDH*) and expressed as fold change (2^−ΔΔCt^) relative to untreated controls, defined as 1. Data are expressed as mean ± SD (* *p* ≤ 0.05; ** *p* ≤ 0.01; *** *p* ≤ 0.001). Experiments were conducted using five independent biological replicates per condition, each analyzed in technical triplicate. Technical replicates were averaged within each biological replicate before statistical analysis.

**Figure 2:**
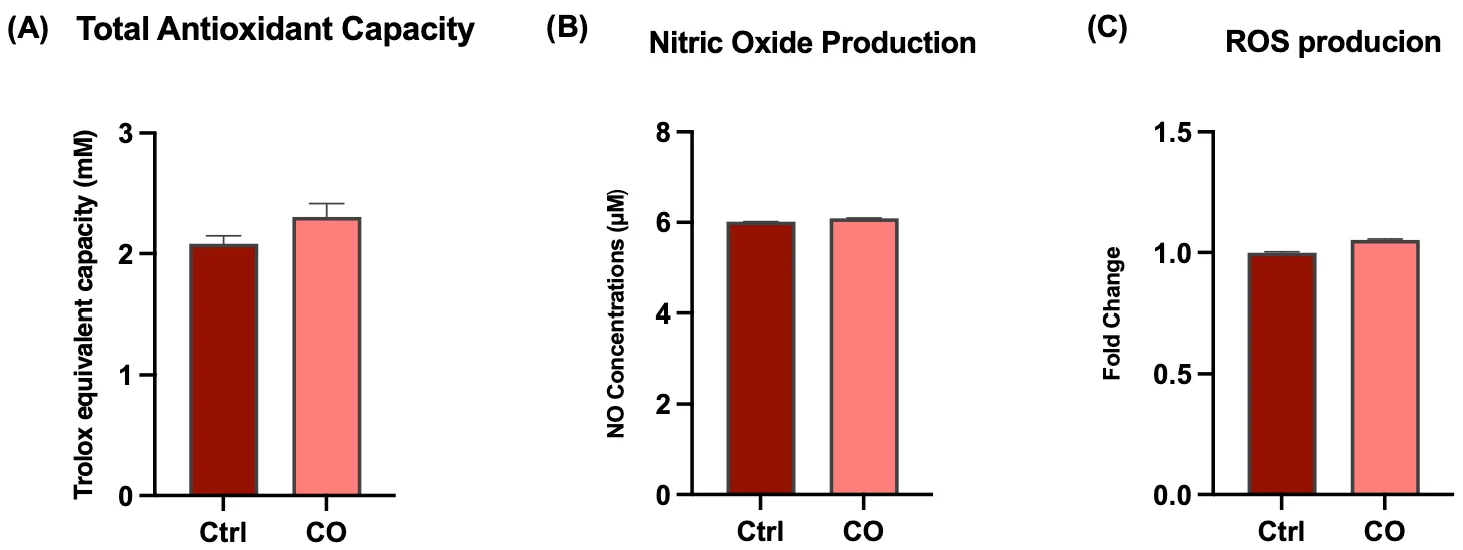
Total antioxidant capacity (**A**), NO-related nitrite levels (**B**), and CM-H2DCFDA fluorescence (**C**) in control vs. REAC CO-IBZ-treated HFF1. Data are expressed as mean ± SD. ROS values are shown relative to untreated controls, whereas total antioxidant capacity and NO-related nitrite levels are reported according to the corresponding assay readouts. Experiments were performed using five independent biological replicates per condition, with technical replicates averaged within each biological replicate before statistical analysis.

**Figure 3:**
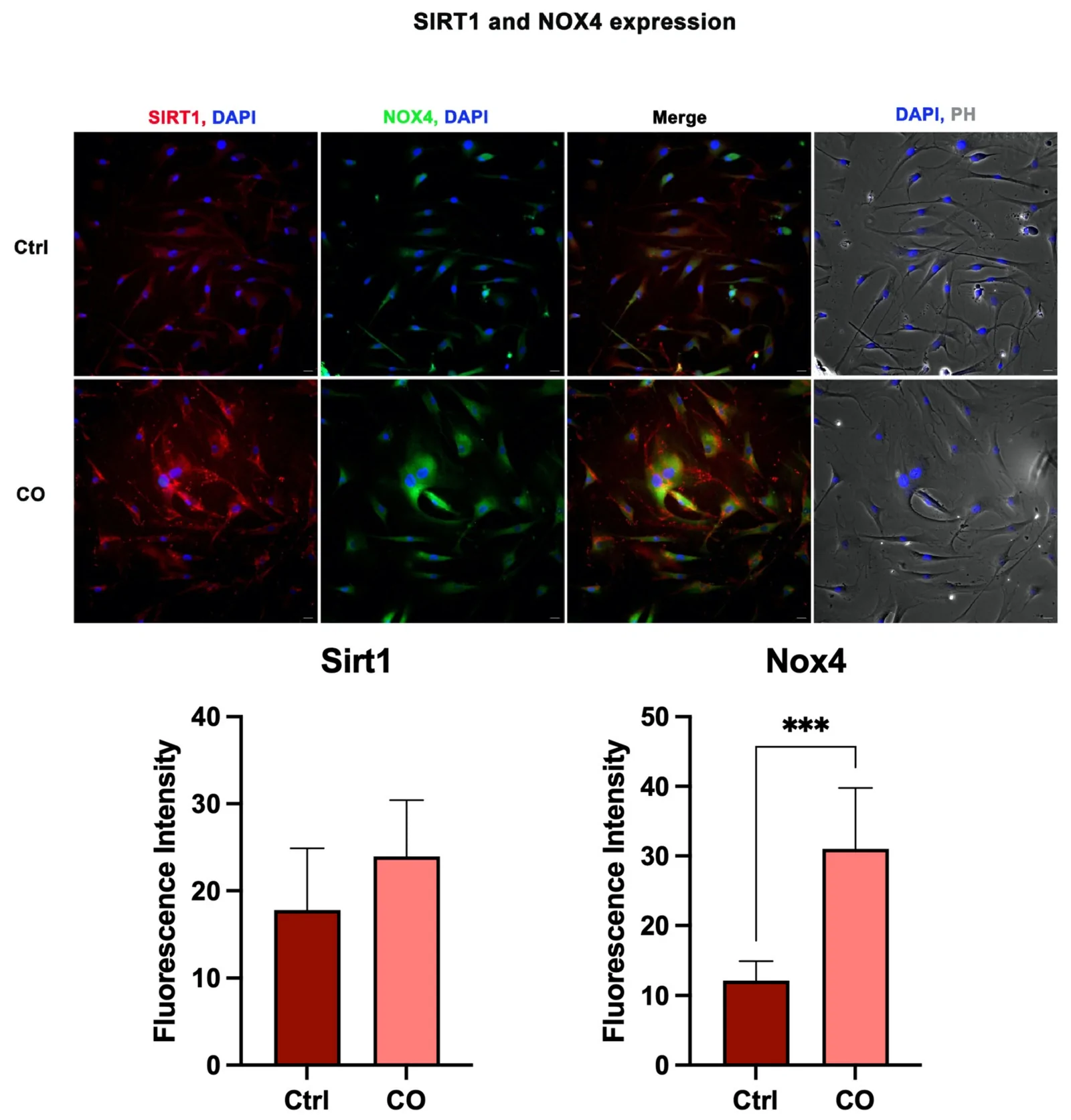
Representative immunofluorescence images and quantitative mean fluorescence intensity analysis of *SIRT1* and *NOX4* in HFF1 cells after REAC CO-IBZ treatment compared with untreated controls. Nuclei are counterstained with 4′,6-diamidino-2-phenylindole (DAPI). Scale bar: 40 μm. Images were acquired using identical exposure and acquisition settings and are shown as representative fields from independent biological replicates. Fluorescence intensity was quantified using ImageJ/Fiji software. Data are presented as mean ± SD (*SIRT1*: ns; *NOX4*: *** *p* ≤ 0.001). Quantitative analyses used *n*= 5 independent biological replicates per condition.

**Figure 4:**
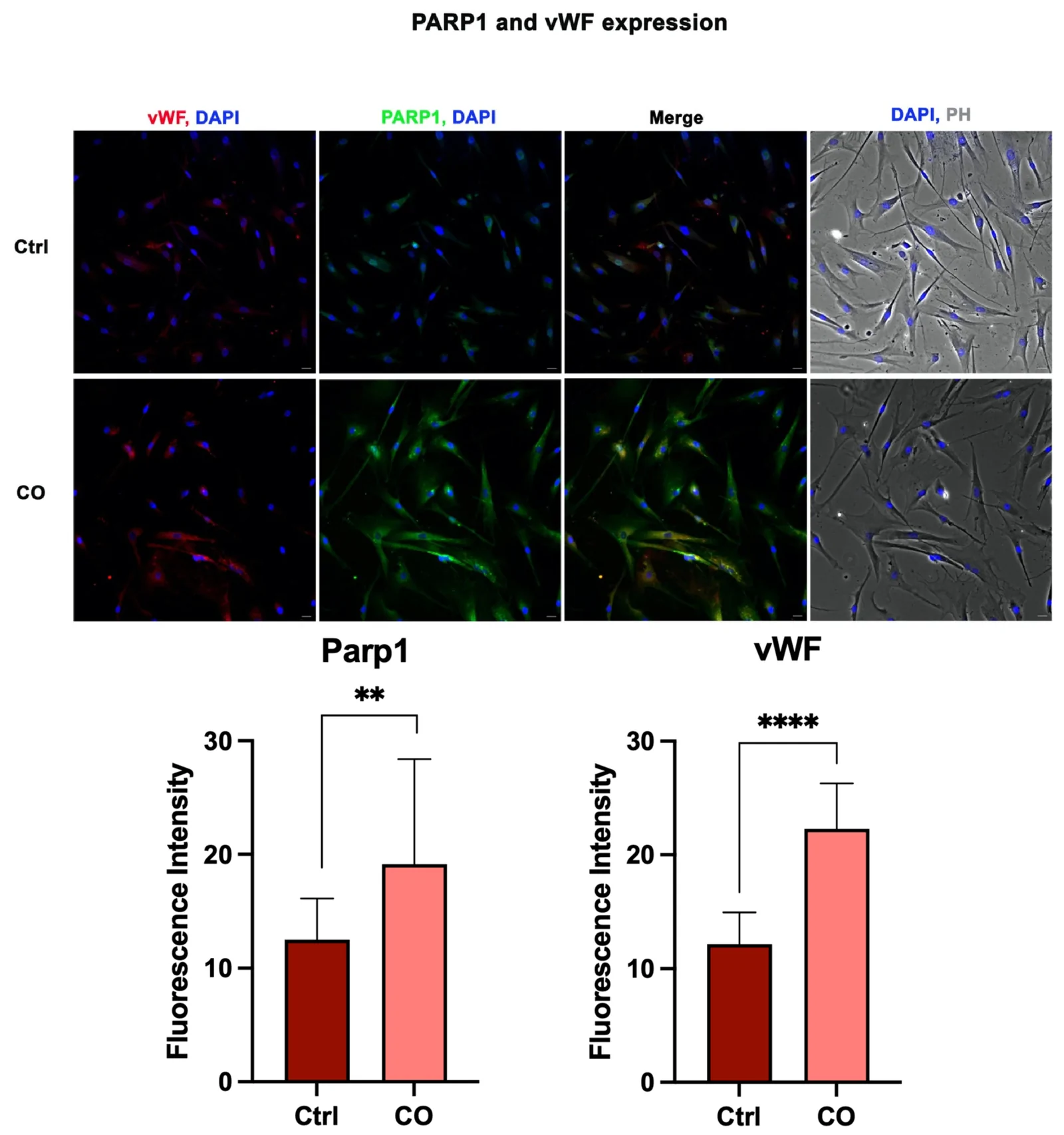
Representative immunofluorescence images and quantitative mean fluorescence intensity analysis of vWF and PARP1 in HFF1 cells after REAC CO-IBZ treatment compared with untreated controls. Nuclei are counterstained with 4′,6-diamidino-2-phenylindole (DAPI). Scale bar: 40 μm. Images were acquired using identical exposure and acquisition settings and are shown as representative fields from independent biological replicates. Fluorescence intensity was quantified using ImageJ/Fiji software. Data are presented as mean ± SD (PARP1: ** *p* ≤ 0.01; vWF: **** *p* ≤ 0.0001). Quantitative analyses used *n* = 5 independent biological replicates per condition.

**Figure 5:**
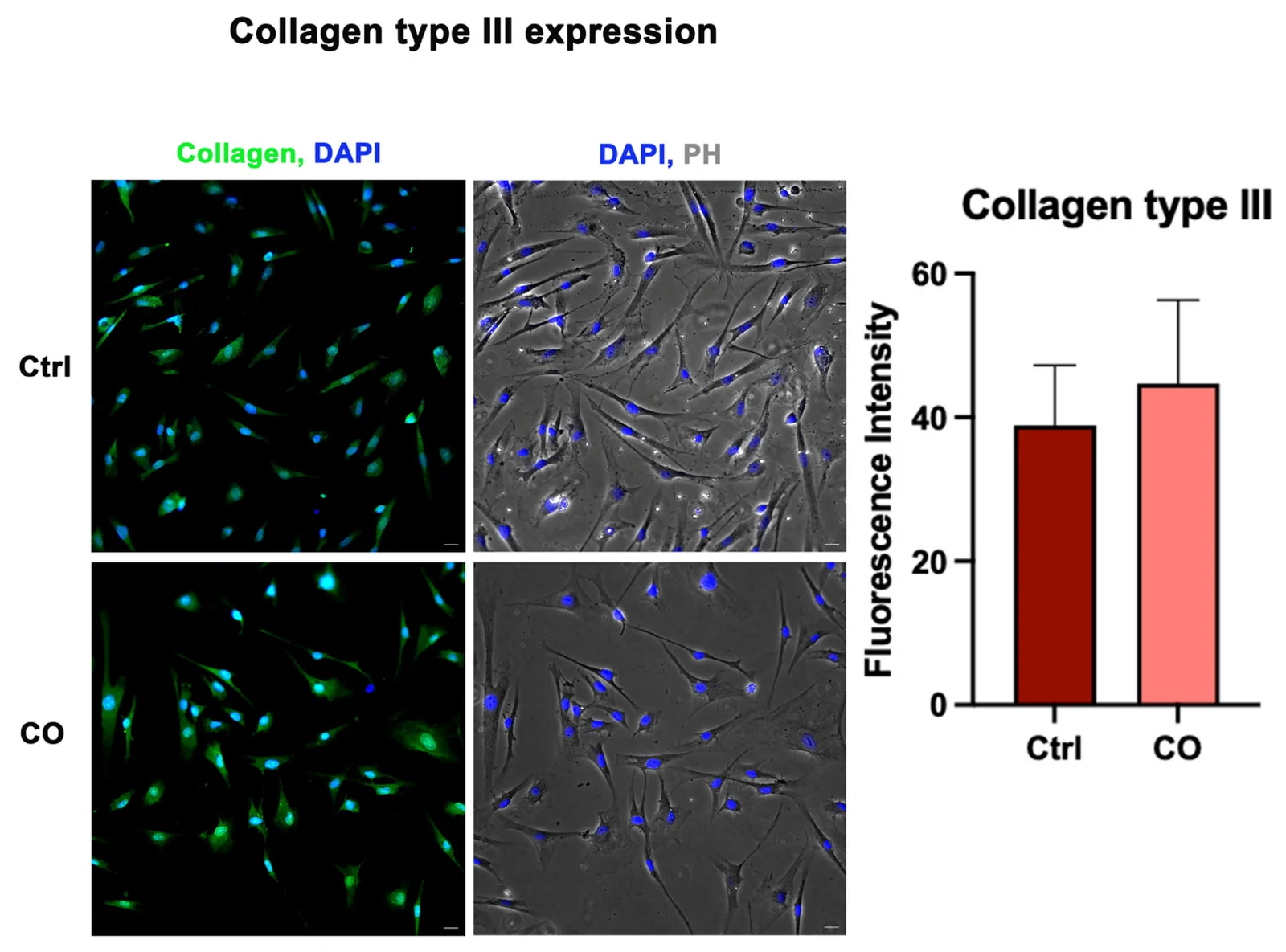
Representative immunofluorescence images and quantitative mean fluorescence intensity analysis of collagen type III in HFF1 cells after REAC CO-IBZ treatment compared with untreated controls. Nuclei are counterstained with 4′,6-diamidino-2-phenylindole (DAPI). Scale bar: 40 μm. Images were acquired using identical exposure and acquisition settings and are shown as representative fields from independent biological replicates. Fluorescence intensity was quantified using ImageJ/Fiji software. Data are presented as mean ± SD (ns). Quantitative analyses used *n* = 5 independent biological replicates per condition.

## Data Availability

The original contributions presented in this study are included in the article. Further inquiries can be directed to the corresponding author.
